# A comparison of four fibrosis indexes in chronic HCV: Development of new fibrosis-cirrhosis index (FCI)

**DOI:** 10.1186/1471-230X-11-44

**Published:** 2011-04-21

**Authors:** Waqar Ahmad, Bushra Ijaz, Fouzia T Javed, Sana Gull, Humaira Kausar, Muhammad T Sarwar, Sultan Asad, Imran Shahid, Aleena Sumrin, Saba Khaliq, Shah Jahan, Asim Pervaiz, Sajida Hassan

**Affiliations:** 1 Applied and Functional Genomics Lab, Centre of Excellence in Molecular Biology, University of the Punjab, Lahore-53700, Pakistan; 2 Fouzia Tahir Javed, Department of Pathology, Jinnah Hospital, Lahore-54590, Pakistan

## Abstract

**Background:**

Hepatitis C can lead to liver fibrosis and cirrhosis. We compared readily available non-invasive fibrosis indexes for the fibrosis progression discrimination to find a better combination of existing non-invasive markers.

**Methods:**

We studied 157 HCV infected patients who underwent liver biopsy. In order to differentiate HCV fibrosis progression, readily available AAR, APRI, FI and FIB-4 serum indexes were tested in the patients. We derived a new fibrosis-cirrhosis index (FCI) comprised of ALP, bilirubin, serum albumin and platelet count. FCI = [(ALP × Bilirubin) / (Albumin × Platelet count)].

**Results:**

Already established serum indexes AAR, APRI, FI and FIB-4 were able to stage liver fibrosis with correlation coefficient indexes 0.130, 0.444, 0.578 and 0.494, respectively. Our new fibrosis cirrhosis index FCI significantly correlated with the histological fibrosis stages F0-F1, F2-F3 and F4 (r = 0.818, p < 0.05) with AUROCs 0.932 and 0.996, respectively. The sensitivity and PPV of FCI at a cutoff value < 0.130 for predicting fibrosis stage F0-F1 was 81% and 82%, respectively with AUROC 0.932. Corresponding value of FCI at a cutoff value ≥1.25 for the prediction of cirrhosis was 86% and 100%.

**Conclusions:**

The fibrosis-cirrhosis index (FCI) accurately predicted fibrosis stages in HCV infected patients and seems more efficient than frequently used serum indexes.

## Background

Hepatitis C virus (HCV) is considered as a major basis of liver associated diseases throughout the world. More than 350 million people (3% of the world's populations) [1,2] and almost 10 million people in Pakistan are infected with HCV [3]. The genotypes 3a, 3b, 1a and 4a are most prevalent in Pakistan [4]. It is predicted that hepatocellular carcinoma (HCC) develops in 1-4% of HCV infected patients in the first five years following the onset of cirrhosis, but cirrhosis may occur with in the range of 10-50 years [5]. In HCV infected patients, liver biopsy is considered essential to stage liver fibrosis. Procedure of liver biopsy is invasive, expensive and not suitable for all patients. Patients can have severe side effects like pain andharsh complications also leading to death [[1,3] and [5]]. Many previous studies reported that host factors reflect fibrosis development leading to HCC [6,7], so these can be used as non-invasive means to overcome the weaknesses arise from biopsy procedures. Chronic hepatitis C is known as hepatic lesions associated with increased levels of aminotransferases more than 6 months. Moreover, treatment with interferon therapy should be based on the liver fibrosis stage [8]. Various authors tried to find accurate non-invasive markers and develop correlations between the serum aminotransferases levels, hyluronic acid level, collagen level, platelet count and HCV viral titer with fibrosis stages but no clear conclusions were formed. Several scoring systems like AST to ALT ratio (AAR), AST-Platelet ratio (APRI), Fibrotest (FT), Fibrosis Index (FI) and FIB-4 with different thresholds to predict presence or absence of fibrosis or cirrhosis in patients infected with HCV had been proposed. However, mild fibrosis (F0) to end stage cirrhosis cannot be predicted accurately using a single system [9-18].

In this study, we compared and evaluated diagnostic accuracy of the readily available non-invasive serum indexes including AAR, APRI, FI and FIB-4 to find accurate and reliable non-invasive markers for evaluating fibrosis progression. We also developed a new non-invasive serum marker index for this purpose by evaluating several clinic-pathological features. A marker with high predictive values would eliminate the need of liver biopsy that also reduces the cost and risks associated to it.

## Methods

### Patients

This study was conducted at the Department of Pathology, Jinnah Hospital, Lahore; Mayo Hospital, Lahore and Liver Centre, Faisalabad in collaboration with Applied and Functional Genomics Lab, National Centre of Excellence in Molecular Biology (CEMB), University of the Punjab, Lahore, Pakistan. HCV RNA-positive patients were identified among HCV antibody (anti-HCV) positive patients. Later, the study plan was discussed with patients and the biopsy was taken only from those patients who were willing for this procedure. The purpose of this study was to design a new Index so that disease progression can be evaluated non-invasively and future need of biopsy can be eliminated. This was a retrospective cross-sectional study. This analytical study was carried out from March 2008 to September 2010.

Patients who received a previous course of INF or immunosuppressive therapy or who had clinical evidence of HBV or HIV and any type of liver cancer were excluded from the study. Patients who refused to have a liver biopsy or for whom it was contraindicated, i.e., because of a low platelet count, prolonged prothrombin time or decompensated cirrhosis were also excluded from the study. The liver biopsy procedure, its advantages and possible adverse effects were explained to the patients. Informed consent were obtained from patients contained information about demographic data, possible transmission route of HCV infection, clinical, virological and biochemical data. This study included 157 patients (M/F 114/43; mean age 38.1 ± 10.2, age range 19-58 years). The study was approved by Institutional Review Board (IRB, CEMB). The Federal-wide Assurance document (ID: FWA00001758) was approved by the local office for Human Research Protection.

### Histological evaluation of biopsy samples

The histological evaluation of paraffin-embedded liver specimens was carried out at the Pathology Department, Jinnah Hospital, Lahore, according to METAVIR scoring system [19]. Liver biopsies were evaluated by two independent pathologists without prior information to patient's history. Histological staging based on the degree of fibrosis have five degrees of fibrosis: as F0 (no fibrosis), F1 (mild fibrosis without septa), F2 (moderate fibrosis with few septa), F3 (severe fibrosis with numerous septa without cirrhosis) and F4 (cirrhosis). We further grouped fibrosis stages as F0-F1 (minimal fibrosis), F2-F3 (advanced fibrosis), F4 (cirrhosis) and F2-F4 (significant fibrosis).

### HCV RNA detection and quantitative PCR

RNA was extracted from 140 μl serum samples using QIAamp viral RNA extraction kit (Qiagen USA cat # 52906) according to the manufacturer's protocol. cDNA was synthesized using Moloney murine leukemia virus (MmLV) reverse transcriptase (Invitrogen, USA). First round and nested PCRs were carried out with Taq Polymerase (Fermentas USA) and analyzed on 2% agarose gel. Qiagen HCV quantitative kit was used to perform HCV RNA quantification with 10 ul of the extracted RNA on Roche Real Time PCR using fluorescent probes to detect amplification after each replicating cycle.

### HCV genotyping

HCV genotyping was carried out using Invader HCV genotyping assay (Third wave technology USA). Briefly, about 100 ng of the HCV RNA was reverse transcribed to cDNA using 200U of MmLV (Invitrogen, USA). From the amplified product, 2 μl was taken and the genotyping assay was performed for 12 different HCV types.

### Comparison of already available non-invasive serum biomarkers to evaluate patient's liver biopsy data

Serum samples and liver specimens collected from each patient were stored at -70°C for further biochemical analysis. The routine liver function tests (LFTs), Hb, serum albumin and direct bilirubin levels were anticipated for each patient. All biochemical tests and their scores were made without knowledge of liver biopsy results and all patients were evaluated for AAR, APRI, Fibrosis Index (FI) and FIB-4 indexes.

The following formulas were used to review the predicted scores with the particular cut-off values as mentioned previously.

• **AAR **[15] = AST (IU/l)/ ALT (IU/l)

If AST/ALT ≥ 1, significant cirrhosis

• **APRI **[16] = [{AST (IU/l)/ ALT_ULN (IU/l)}× 100]/ platelet count (10^9^/l)

If APRI < 0.5, no or minimal fibrosis; if APRI > 1.5, significant fibrosis

• **FI **[17] = 8.0 - 0.01 × PLT (10^9^/l) - serum albumin (g/dl)

If F-Index < 2.1, no or minimal fibrosis; F-index ≥ 2.1, significant fibrosis, and if F-Index ≥ 3.3, significant cirrhosis

• **FIB-4 **[18] = [Age (Years) × AST (IU/l)]/[Platelet count (× 10^9^/l) × ALT (IU/l)1/2]

If FIB-4 < 1.45, no or minimal fibrosis, If FIB-4 > 3.25, significant fibrosis

### Statistical analysis

The data was analyzed using statistical package SPSS version 16 for windows. A *p *value of 0.05 was considered statistically significant. All data was presented as mean values or no. of patients. Spearman's rank correlation was used to assess the significant association between continuous variables and liver fibrosis stages. The student *t*-test was used to compare arithmetic means and parameters while Chi-square (*X*2) test was used to compare categorical data, correlation with Fisher's exact test was used when appropriate. Patients were divided into three main groups as, patients with no or minimal fibrosis (F0-F1), patients with significant fibrosis (F2-F3) and patients with clinically significant cirrhosis (F4). The independently distinguished values of biochemical markers and AAR, APRI, FIB-4 and FI indexes for the prediction of significant fibrosis and cirrhosis were evaluated using univariate and multiple regression analysis. Area under the receiver operating characteristic (ROC) curves (AUROCs) was used to compare and deduce the diagnostic accuracies of the selected bio-markers.

## Results

### Patient's data

The demographic and clinical outcomes of the 157 HCV infected patients are briefly explained in Table 1. The evaluation of chronic HCV activity (inflammatory grade) showed mild chronic hepatitis in 51 patients, moderate chronic hepatitis in 67 patients and severe chronic hepatitis in 39 patients. The determination of liver fibrosis showed stage F0 in 29, F1 in 39, 34 patients in F2 and F3 stage each and 21 patients in F4 or advanced fibrosis leading to cirrhosis. Our data showed the presence of genotype 1a in 22 and 3a in 135 patients, 95 patients were < 40 years of age, while 62 were > 40 years of age.

**Table 1:** Demographic, clinical, and liver histological features of 157 chronic HCV infected patients

Features	Mean (± SD)	Minimum	Maximum
Sex (Male/Female)	114/43	-	-
Age (years)	38.1 ± 10.2	19	58
Age groups (< 40/ > 40)	95/62	-	-
Genotype (1a/3a)	22/135	-	-
Viral load (IU/ml)	5.47 × 10^7^ ± 1.4 × 10^8^	1.7 × 10^4^	1.01 × 10^9^
Hb level (g/dl)	12.7 ± 1.3	10.3	16.7
Bilirubin (mg/dl)	1.03 ± 0.36	0.5	2.1
ALT (IU/l)	134.4 ± 63.1	18	271
ALP (IU/l)	147.7 ± 101.5	20	438
AST (IU/l)	106.7 ± 68.2	20	395
Serum Albumin (g/dl)	4.1 ± 0.31	3.1	4.9
Platelet count (× 10^9^/l)	154.5 ± 41.7	49	229

**Liver fibrosis stages, n (%)**			

F0	(n = 29, 18.5%)	-	-
F1	(n = 39, 24.8%)	-	-
F0+F1 (minimal fibrosis)	(n = 68, 43.3%)	-	-
F2	(n = 34, 21.7%)	-	-
F3	(n = 34, 21.7%)	-	-
F2+F3 (advanced fibrosis)	(n = 68, 43.3%)	-	-
F4 (Cirrhosis)	(n = 21, 13.4%)	-	-
F2+F3+F4 (significant fibrosis)	(n = 89, 56.7%)	-	-

### Relationship between clinical findings and fibrosis

Liver fibrosis stages were statistically significant between age groups (*p*< 0.05). Mild and moderate fibrosis was diagnosed mostly in younger patients while more advanced stages were observed in patients over 40 years old. Patients with F0 fibrosis were too young as compared to those who developed moderate or severe fibrosis leading to cirrhosis (Mean age ± SD, 25.9 ± 2.4 years). The distribution of liver fibrosis stages with regard to gender and genotypes of patients illustrated in Table 2 showed no significant differences (for gender: *p = 0.247 *and for genotypes: *p = 0.258*). Univariate analysis revealed that serum viral loads, bilirubin, albumin, platelet count, AST and ALP levels were significantly different in various fibrosis stages (Table 2).

**Table 2:** Distribution of each variable according to fibrosis stages

Factor	F0 (n = 29)	F1(n = 39)	F2(n = 34)	F3(n = 34)	F4(n = 21)	*P *value
Age	25.9 ± 2.4	37.9 ± 9.5	42.8 ± 7.6	37.7 ± 8.5	48.4 ± 7.1	< 0.05
Sex (M/F)	19/10	35/4	26/8	20/14	14/7	0.247
Genotype (1a/3a)	8/21	4/35	3/31	3/31	4/17	0.258
HAI score	3.8 ± 2.1	6.05 ± 2.8	6.8 ± 3.2	6.9 ± 4.02	7.6 ± 2.9	< 0.05
Viral load	6.2 × 10^5^ ± 1.2 × 10^6^	1.3 ± × 10^7^ ± 1.5 × 10^7^	2.1 × 10^8^ ± 2.4 × 10^8^	2.4 × 10^7^ ± 2.5 × 10^7^	2.9 × 10^5^ ± 2.9 × 10^5^	< 0.05
Hb level	12.8 ± 1.3	12.6 ± 1.2	12.77 ± 1.7	12.9 ± 1.3	12.3 ± 1.2	0.328
Bilirubin	0.7 ± 0.1	0.88 ± 0.2	0.99 ± 0.27	1.2 ± 0.3	1.62 ± 0.31	< 0.05
ALT	120.2 ± 71.9	117.8 ± 55.3	152.1 ± 66.5	139.6 ± 56.6	147.5 ± 61.2	0.091
ALP	72.6 ± 16.8	88.1 ± 47.5	110.7 ± 45.1	208.6 ± 75.9	323.8 ± 80.1	< 0.05
AST	83.1 ± 57.9	107.1 ± 66.5	95.9 ± 51.4	106.9 ± 65.4	155.5 ± 90.6	< 0.05
Albumin	4.5 ± 0.1	4.3 ± 0.16	4.2 ± 0.18	4.08 ± 0.2	3.6 ± 0.33	< 0.05
Platelet count	191.3 ± 18.7	185.1 ± 21.2	162.1 ± 16.6	125.5 ± 18.7	81.6 ± 17.7	< 0.05
AAR	0.8 ± 0.58	1.14 ± 1.21	0.93 ± 1.1	1.02 ± 1.03	1.26 ± 0.95	0.522
APRI	1.04 ± 0.75	1.39 ± 0.87	1.40 ± 0.75	2.09 ± 1.33	4.7 ± 3.1	< 0.05
FI	1.24 ± 0.26	1.51 ± 0.28	1.81 ± 0.27	2.33 ± 0.28	3.2 ± 0.4	< 0.05
FIB-4	1.21 ± 0.95	2.17 ± 1.47	2.31 ± 1.57	3.06 ± 2.1	8.73 ± 6.59	< 0.05

### Diagnosis of fibrosis using already available AAR, APRI, F-Index, and FIB-4 serum indexes

**Figure 1:**
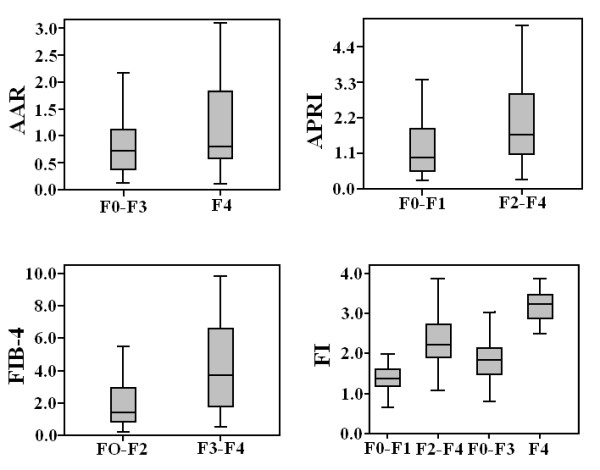
**Box plots of the AAR, APRI, FIB-4 and FI for different fibrosis stages**. The horizontal line inside each box represents the median, while the top and bottom of boxes represent the 25^th ^and 75^th ^percentiles, respectively. Vertical lines from the ends of the box encompass the extreme data points.

The relationship between the fibrosis stages and four serum indexes: AAR, APRI, FI and FIB-4 is illustrated in Figure 1 (see also Table 2). There was a significant relationship between fibrosis stages and serum indexes except AAR (*p *> 0.05). A gradual increase in the level of APRI, FI and FIB-4 indexes was observed in fibrosis stages.

**Figure 2:**
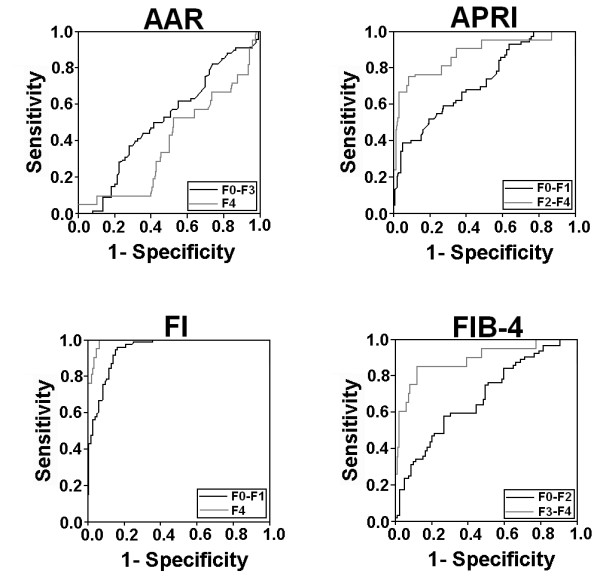
**Receiver operating characteristic curves generated by four serum markers, AAR, APRI, FIB-4 and FI for differentiation between patients in fibrosis stage F0-F1, F2-F3 and F4**.

The AUROCs of the serum non-invasive indexes scores are shown in Table 3. AUROC of FI was higher than APRI (*p*< 0.05) for differentiating minimal fibrosis from significant fibrosis (Figure 2). To predict cirrhosis, FI showed high AUROC than AAR. Spearman correlation between each serum index score and fibrosis stages was high for F-Index, FIB-4 and APRI, while, AAR showed significantly low 'r' index indicated in Table 3. By using published cut-off values for each index, we analyzed the sensitivity and specificity of each index for significant fibrosis and cirrhosis. Patients with minimal fibrosis can be identified from advanced/significant or cirrhotic patients using FIB-4, AAR, APRI and F-Index with sensitivity 51%, 67.6%, 19.1% and 100% and specificity 85.4%, 42.8%, 97.7% and 58.4%, respectively. At a cut-off value > 3.25 for FIB-4, > 1.5 for APRI, > 1 for AAR and > 3.3 for F-Index have 59.2%, 34.8%, 42.8% and 38.1% sensitivity and 82.3%, 67.6%, 67.6% and 100% specificity, respectively, to discriminate advanced fibrosis stages from minimal.

**Table 3:** Validity of serum AAR, APRI, FIB-4 and FI in 157 HCV infected patients

AAR							
**Cutoff value**	**Spe%**	**Sen%**	**PPV%**	**NPV%**	**F0-F3 (n = 136 )**	**F4 (n = 21)**	**AUC [95% CI]**

< 1	42.8	67.6	88.4	16.9	92/44	12/9	0.468[0.377-0.559]
> 1	67.6	42.8	16.9	88.4	44/92	9/12	0.610[0.483-0.738]

**APRI**							

**Cutoff value**	**Spe%**	**Sen%**	**PPV%**	**NPV%**	**F0-F1 (n = 68)**	**F2-F4 (n = 89)**	**AUC [95% CI]**

< 0.5	97.7	19.1	86.6	61.2	13/55	2/87	0.715[0.635-0.795]
> 1.5	67.6	34.8	58.4	44.2	22/46	31/58	0.876[0.782-0.971]

**FIB-4**							

**Cutoff value**	**Spe%**	**Sen%**	**PPV%**	**NPV%**	**F0-F2 (n = 102)**	**F3-F4 (n = 55 )**	**AUC [95% CI]**

< 1.45	85.4	51	86.6	48.4	52/50	8/47	0.732 [0.655-0.809]
> 3.25	82.3	59.2	64	79.2	18/84	33/22	0.545[0.456-0.635]

**FI**							

**Cutoff value**	**Spe%**	**Sen%**	**PPV%**	**NPV%**	**F0-F1 (n = 68)**	**F2-F4 (n = 89)**	**AUC [95% CI]**

< 2.1	58.4	100	64.7	100	68/0	37/52	0.939[0.903-0.974]

**Cutoff value**	**Spe%**	**Sen%**	**PPV%**	**NPV%**	**F0-F3 (n = 136)**	**F4 (n = 21)**	**AUC [95% CI]**

> 3.3	100	38.1	100	91.2	0/136	8/13	0.990[0.979-1.001]

Spearman rank correlation coefficient (R-index) for AAR = 0.130, APRI = 0.444, FIB-4 = 0.494 and FI = 0.578

### Diagnosis of fibrosis with clinic-pathological features including viral load, Hb level, bilirubin, ALT, ALP, AST, albumin and platelet count

**Figure 3:**
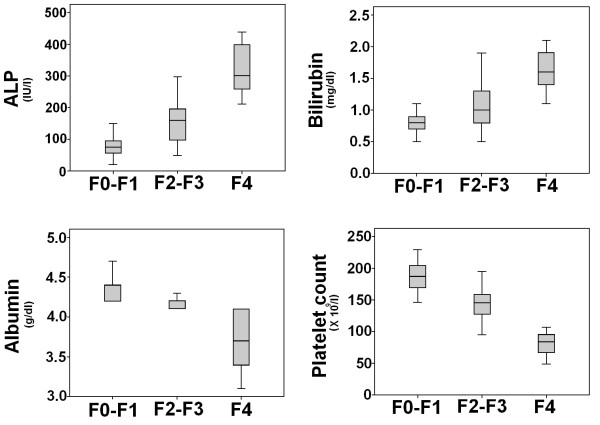
**Relationship between fibrosis stages and the ALP, bilirubin, serum albumin and fibrosis-cirrhosis index (FCI). **The lines through the middle of the boxes represent the median, while the top and bottom of the boxes are the 25^th ^and 75^th ^percentiles. The error bars represent measurement range (maximum and minimum values).

Viral load was significant among fibrosis stages. It gradually increased in advanced fibrosis, and then suddenly dropped in cirrhosis. ALT and Hb levels were not significant, while AST levels were noteworthy to differentiate liver fibrosis stages. Meanwhile, only 16 (10.1%) and 21 (13.3%) patients showed normal ALT and AST levels, respectively, independent of fibrosis stage. The discriminative values of the biochemical markers for the prediction of different fibrosis stages were determined by logistic regression analysis. By univariate analysis (*p *< 0.05, Table 2), viral load, bilirubin, ALP, AST, albumin and platelet count were significantly associated with various fibrosis stages. However, in multivariate analysis, bilirubin, ALP, albumin and platelet count were found to be independently predictive (Table 4). This information related to these biochemical markers can also be helpful in differentiating liver fibrosis stages. Figure 3 shows the box plot of these four markers with liver histological stages. It is clear from Figure 3 and Table 2 that as the fibrosis increased, bilirubin and serum ALP level also increased, while platelet count and albumin level gradually reduced in cirrhosis. It was interesting to note that serum ALP and bilirubin was 2 times and 5 times higher in cirrhotic patients, respectively, than normal limits.

**Table 4:** Multivariate analysis of ALP, bilirubin, albumin and platelet count for discrimination between F0-F1 and F2-F3, and F2-F3 and F4 patients

Variables	F0-F1/F2-F3		F2-F3/F4	
	
	OR (95% CI)	*P *value	OR (95% CI)	*P *value
ALP	1.5 (105.13-51.34)	< 0.05	1.3 (203.25-124.93)	< 0.05
Bilirubin	2.1 (0.399- 0.122)	< 0.05	1.4 (0.719- 0.403)	< 0.05
Albumin	1.1 (0.126- 0.308)	< 0.05	1.3 (0.333- 0.598)	< 0.05
Platelet count	1.2 (34.71- 53.35)	< 0.05	1.2 (48.63- 75.72)	< 0.05

**Figure 4:**
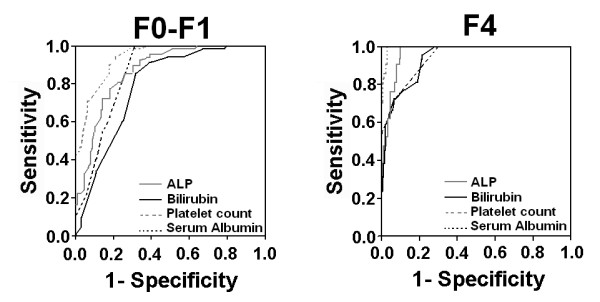
**Receiver operating characteristic curves for individual serum markers; ALP, bilirubin, platelet count and serum albumin for the predication of F0-F1, F2-F3 and F4 fibrosis stages**.

Based on ROC curve analysis as illustrated in Figure 4, four significant serum markers ALP, bilirubin, albumin and platelet count showed superior diagnostic power with high AUROCs for differentiating various fibrotic stages and cirrhosis as given in Table 5. Our data showed that if these four serum markers ALP, bilirubin, albumin and platelet count are used simultaneously, they have high PPV and NPV for predicting cirrhosis and differentiating no/minimal fibrosis from significant fibrosis.

**Table 5:** Diagnostic accuracy of the ALP, bilirubin, albumin, platelet count and fibrosis-cirrhosis index (FCI) for the prediction of F0-F1 stage and hepatic cirrhosis in chronic HCV infected patients (n = 157)

Marker Cutoff	Interpretation		AUC [95% CI]	Spe%	Sen%	PPV%	NPV%
**ALP**							

< 120	F0-F1 (n = 68)	F2-F4 (n = 89)	0.829 [0.760-0.897]	69.6	85.2	68.2	86.1
						
	58/10	27/62					
					
> 240	F4 (n = 21)	F0-F3 (n = 136)	0.931 [0.882-0.981]	91.9	80.9	60.7	96.9
						
	17/4	11/125					

**Bilirubin**							

< 0.95	F0-F1 (n = 68)	F2-F4 (n = 89)	0.729 [0.643-0.815]	68.1	85.2	67.4	85.7
						
	58/10	28/60					
					
> 1.5	F4 (n = 21)	F0-F3 (n = 136)	0.889 [0.816-0.962]	95.5	66.6	70	94.8
						
	14/7	6/130					

**Albumin**							

> 4.1	F0-F1 (n = 68)	F2-F4 (n = 89)	0.812 [0.738-0.886]	67.4	100	70.1	100
						
	68/0	29/60					
					
< 3.85	F4 (n = 21)	F0-F3 (n = 136)	0.879 [0.796-0.962]	92.6	71.4	60	95.4
						
	15/6	10/126					

**Platelet count**							

> 150	F0-F1 (n = 68)	F2-F4 (n = 89)	0.935[0.900-0.970]	69.6	98.5	71.2	98.4
						
	67/1	27/62					
					
< 100	F4 (n = 21)	F0-F3 (n = 136)	0.990[0.977-1.002]	98.5	80.9	89.4	97.1
						
	17/4	2/134					

**FCI**							

< 0.130	F0-F1 (n = 68)	F2-F4 (n = 89)	0.932[0.895-0.969]	86.5	80.8	82.1	85.5
						
	55/13	12/77					
					
> 1.25	F4 (n = 21)	F0-F3 (n = 136)	0.996[0.989-1.002]	100	85.7	100	97.8
						
	18/3	0/136					

Spearman rank correlation coefficient (R-index) for ALP = 0.716, bilirubin = 0.597, albumin = -0.700, platelet count = -0.817 and FCI = 0.818.

For the detection of significant cirrhosis, platelet count less than 100 showed 81% sensitivity, 98% specificity, 89% PPV and 97% NPV. For the same outcome, ALP > 240 IU/l had sensitivity, specificity, PPV and NPV of 90%, 92%, 60.7% and 97%, respectively. The bilirubin and albumin were also quite sensitive for the presence of cirrhosis. Bilirubin level > 1.5 had a sensitivity 66.6%, specificity 95.5%, PPV 70% and NPV 94%, while albumin < 3.85 g/dl has sensitivity, specificity, PPV and NPV 71.4%, 93%, 60% and 95%, respectively.

In no/minimal fibrosis, ALP < 120 IU/l showed sensitivity, specificity, PPV and NPV 85%, 70%, 68% and 86%, respectively. At cut-off value > 150, platelet count also showed high sensitivity (98%) and specificity (70%) with 71.2% PPV and 98% NPV. Serum bilirubin and albumin also showed same pattern with high sensitivity, specificity, PPV and NPV as shown in Table 5.

### Construction of a new Index for the prediction of fibrosis stage

Based on the relationship of the regression coefficients of four-biochemical markers, ALP, bilirubin, albumin and platelet count, we developed a new fibrosis-cirrhosis index for the prediction of HCV disease progression from initial fibrosis stage to end stage cirrhosis.

It can be represented as

**Figure 5:**
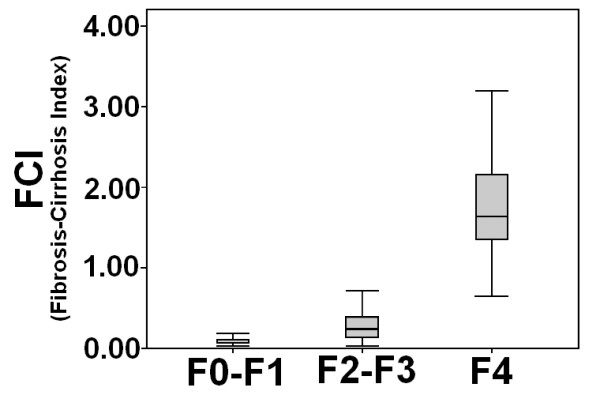
**Box plot of fibrosis-cirrhosis index (FCI) for each fibrosis stage**. The horizontal line inside each box represents the median, while the top and bottom of boxes represent the 25^th ^and 75^th ^percentiles, respectively. Vertical lines from the ends of the box encompass the extreme data points.

**Figure 6:**
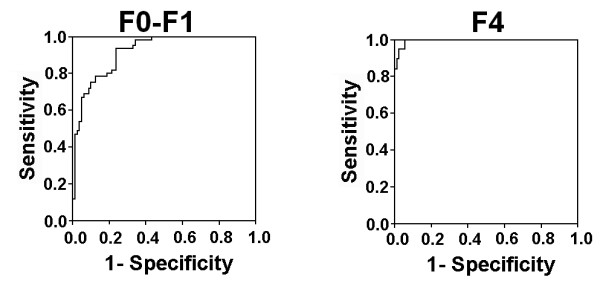
**Receiver operating characteristic curves generated by the fibrosis-cirrhosis index (FCI) to discriminate fibrosis stages F0-F1 and F4**. FCI showed maximum AUC for prediction of F4 (cirrhosis).

The FCI distribution for the patients in the respective fibrosis stages is represented in Figure 5. The median values for FCI in F0-F1, F2-F3 and F4 patients were 0.085, 0.32 and 1.9, respectively. FCI significantly correlated with the liver fibrosis stages (Spearman's rank correlation coefficient, r = 0.818, *P*< 0.05). The diagnostic values of F1 to differentiate F0-F1 and F4 patients were evaluated using the AUROCs (Figure 6). The AUC for F0-F1 and F4 was 0.932 (CI: 0.895-0.969) and 0.996 (CI: 0.989-1.002), respectively. The cutoff values obtained from the respective ROC curves were < 0.130 and > ≥1.25 in discriminating F0-F1 and F4 patients, respectively. Table 5 illustrates the diagnostic accuracy of FCI. Using a cutoff value of < 0.130, FCI had a sensitivity of 81%, PPV of 82% also with a specificity of 87% and NPV of 82% for the prediction of F0-F1. On the other hand, at a cutoff value of 1.25 or more, FCI had a sensitivity of 86%, specificity and PPV of 100% and 98% NPV for the prediction of cirrhosis (F4).

## Discussion

Hepatocellular carcinoma and hepatic cirrhosis are consequences of chronic hepatitis C. The mean infection time to onset of cirrhosis is approximately 30 years, but cirrhosis may occur within a range of 10-50 years [20]. Fibrosis and its extension in hepatic tissue is most common evidence of cirrhosis. Several indexes are available to predict cirrhosis but no method or score is available on exclusive basis to diagnose earlier fibrosis stages.

Genotype 3a is the most common one followed by 1a in Pakistan [[3,21], and [22]] and same was also observed in this study. Almost, 86% patients had genotype 3a while remaining 14% had genotype 1a. A recent study also reported high prevalence of genotype 3 in HCC patients in Pakistan [23]. Patients with none or initial stage (F0-F1) of fibrosis showed a remarkable difference of age with advanced stages (F2 and F3) of fibrosis and cirrhosis. Most patients with age more than 40 years showed severe fibrosis and cirrhosis. These results confirmed the previous studies that patients with mild fibrosis stage were younger than the moderate and severe disease grade and stage is independent of gender [24-26].

Our results showed positive correlation of ALT with APRI and FI, and negative correlation with AAR and platelet, however, no correlation was established between ALT levels with disease severity and fibrosis stages. Our observation is in agreement with previous reports that serum ALT levels do not accurately predict the presence of hepatic liver damage [27,28]. Several authors reported persistently normal ALT levels (< 42 IU/l) in patients with chronic HCV. Almost, 30% of patients with chronic HCV infection reflect steadily normal serum ALT levels [29-32], however, in our data only 10% (n = 16) patients showed normal ALT levels.

Our data showed gradual increase in serum ALP and bilirubin levels (Table 2) in fibrosis stages when compared to early infection. Both ALP and bilirubin showed strapping significant correlation with disease progression. These results lead them to an important predictor of disease severity. An increased ALP is usually associated with liver metastasis, extraheptic bile obstruction, intraheptic cholestasis, infiltrative liver disease and hepatitis [33,34]. According to Lee *et al*, elevated serum ALP levels were common in liver abscess patients [35]. High bilirubin levels are associated with liver metastases and liver tumor involvement leading to hepatocellular carcinoma and liver cirrhosis by active or non-active HCV or HBV [36]. Limited literature is available on the role of elevated ALP and bilirubin levels in liver fibrosis stages. However, according to Imbert-Bismut *et al. *[37], bilirubin may be used as marker of liver injury, while a change in ALP levels greater than 120 U/L can be indicative of advanced disease progression [12]. These findings suggest that serum ALP and bilirubin may be used as serum markers to assess the disease progression and fibrosis stages in chronic HCV patients.

Many studies supported that platelet count alone may be clinically valuable as a non-invasive serum marker for liver fibrosis and cirrhosis [38,39]. Platelets not only predict fibrosis but also correlate with fibrotic stages [40-42]. Lackner *et al*, [43] showed high AUROC of 0.89 for predicting cirrhosis at platelet value < 150 × 10^9^/L and AUROC of 0.71 for non-cirrhotic patients at a cutoff value > 150 × 10^9^/L. Our data is also in accordance with these results as platelet count showed high AUROC (≥ 0.900) to differentiate different liver fibrosis stages as given in Table 5 and Figure 4. In our study, platelet count was significantly low in cirrhotic patients. At a cutoff value of platelet, < 100 × 10^9^/L has an AUROC of 0.990 for prediction of cirrhosis with 81% sensitivity and 98% specificity. Ginnani *et al*, reported platelet < 130 × 10^9^/L for prediction of cirrhosis in HCV patients with 91.1% sensitivity, 88.3% specificity, PPV 81.2% and NPV 94.7% [44].

We also examined the ability of AAR, APRI, FIB-4 and F-Index for staging liver fibrosis and to differentiate them from cirrhosis. Giannini *et al*, reported a high diagnostic accuracy of AAR > 1.16 with 81.3% sensitivity and 55.3% specificity for the prediction of cirrhosis [44]. However, AAR was not able to differentiate among liver fibrosis stages in our sample data. At value of > 1.0, AAR has 43% sensitivity and 70% specificity for differentiating fibrosis from cirrhosis (Table 3). This poor performance of AAR is similar to that reported by Lackner *et al *[43].

We observed comparatively high APRI (1.24 ± 0.8) and FIB-4 (1.76 ± 1.35) values in F0-F1 patients. The group F0-F1 contains two subgroups, patients with no fibrosis (F0) and with minimal fibrosis (F1). The mean value of APRI and FIB-4 in F0 was 1.04 and 1.21, and in F1 1.39 and 2.17, respectively (Table 2). It is reported that APRI < 0.42 predict mild fibrosis and APRI > 1.2, significant fibrosis in HCV patients with 90% NPV for absence of fibrosis and 91% PPV for fibrosis presence [45-47]. Our results showed that APRI > 1.5 could predict fibrosis with 55% sensitivity, 67% specificity. Moreover, by using same cutoff value of APRI > 1.5 in a recent study by Macias *et al *[48], found that it has 28% sensitivity, 92% specificity, 79% PPV and 55% NPV for predicting significant fibrosis, and for absence of fibrosis APRI < 0.5 has 78%, 44%, 59% and 66% sensitivity, specificity, PPV and NPV, respectively.

FIB-4 was developed by Sterling *et al *in 2006 for diagnosis of fibrosis and cirrhosis in HIV/HCV co-infected patients. We examined this index only for HCV infected patients. A cutoff value of < 1.45 FIB-4 has a NPV for the exclusion of advanced fibrosis of 90%, while a cutoff value > 3.25 has a PPV for the diagnosis of extended fibrosis of 65% [49]. At a cutoff value of < 1.45, Vallet-Pichard observed a high NPV of 94.7% with a sensitivity of 74.3% to exclude severe fibrosis. Where as, for confirming the presence of advanced fibrosis at cutoff value > 3.25, FIB-4 had a PPV of 82.1% with specificity of 98.2% [18]. Our results are not in agreement with Sterling or Vallet-Pichard, as we observed a low NPV (70%) for excluding significant fibrosis, however, we detected a PPV of 83% with specificity of 45% for the presence of advanced fibrosis at cutoff value > 3.25. Trang *et al *[50], proposed new cutoff values of FIB-4 ≤ 1.39 for F0-F1 and ≥2.05 for F2-F4 stage in HCV/HIV co infected patients. At these cutoffs, we observed sensitivity 52%, specificity 76%, PPV 63% and NPV 68% for no/minimal fibrosis and 60%, 63%, 68% and 55% for advanced fibrosis, respectively. Although, we observed low statistical values, our results were in accordance to advance stage prediction. The cut off values proposed by Trang *et al *better predict fibrosis stages in co infected patients and we applied on only HCV infected patients.

Fibrosis index (FI) showed high sensitivity, specificity, PPV, NPV and AUROC for discriminating different fibrosis stages. Ohta developed this simple index in 2006. At cutoff value < 2.1 FI showed sensitivity and specificity for predicting F0-1 stage 66.8% and 78.8% in initial cohort and 68.5% and 63.6% in validation cohort, respectively [17]. At same cutoff, our data showed 100% sensitivity and 58.4% specificity with AUROC 0.939 for the prediction of none/minimal fibrosis. While for predicting cirrhosis in HCV patients, FI value > 3.30 has sensitivity and specificity 67.7% and 75% in initial cohort, and 70.8% and 81% in validation cohort, respectively. However, at this value we observed 33% sensitivity and 100% specificity for predicting cirrhosis (Table 3). We proposed that a new cutoff value of FI > 2.5 can better predict cirrhosis with 95.2% sensitivity and 94% specificity.

The readily available indexes are associated with some limitations like population discrepancy, not able to distinguish all fibrosis stages individually or some primarily developed for co-infected patients. So there is a need to develop a new index that can distinguish minimal fibrosis (F0-F1) from significant (F2-F4) and advanced (F2-F3) from cirrhosis (F4). While considering substantial relationship of routinely applied tests; serum ALP, ALT, AST, Hb level, bilirubin, albumin and platelet count with liver fibrosis stages, we found that four serum markers ALP, bilirubin, albumin and platelet count have high potential to differentiate different fibrosis stages and cirrhosis at given cutoff values (Table 5 and Figure 4). We also observed that combination of these serum markers could better differentiate among fibrosis stages with high sensitivity, specificity, PPV and NPV.

Our newly derived index FCI showed better performance for discriminating between fibrosis stages as compared to AAR, APRI and FI. In initial cohort, the AUROC for predicting F0-F1 stage for FCI was 0.932 when compared to recently used non-invasive serum markers like AAR (AUROC = 0.570) [15], APRI (AUROC = 0.880) [16], FI (AUROC = 0.741) [17], FIB-4 (AUROC = 0.793) [18], Forn's index (AUROC = 0.860) [51], and Fibrotest (AUROC = 0.870) [52]. Moreover, FCI (AUROC = 0.996) showed better performance for predicting cirrhosis than above mentioned serum indexes. Although in our study, platelet count showed high AUROC to predict fibrosis stages, systematic literature reviews consistently shown that panel of fibrosis markers are more accurate than single marker. Combination of two or more serum markers in a mathematical algorithm provide better chance of predicting phase of disease progression instead of individual one [37,53-57]. This analysis showed that FCI has tendency to reflect respective fibrosis stages from no/minimal to cirrhosis with great accuracy (Table 5, Figure 5 and 6). However, several studies are needed to verify these results. Secondly, because of poverty and fear of biopsy, we are not yet able to get enough patient data for verification of our FCI results in new cohort.

## Conclusions

For Pakistani population the mostly used markers were failed to predict fibrosis stages in patients with HCV with accuracy. This study concluded that a simple index (FCI) containing ALP, bilirubin, albumin and platelet count may accurately classify different fibrosis stages from none to cirrhosis. Future studies are required to assess the applicability of this fibrosis-cirrhosis index within different populations and in patients with HBV or other fatty liver diseases.

## Abbreviations

HCV: hepatitis C.

## Competing interests

The authors declare that they have no competing interests.

## Authors' contributions

WA and BI contributed equally to this study. WA, BI and SH designed the study, analyze the data and wrote paper. JS, KS, PA, SA, SG, MTS and IS performed all lab work. FTJ and SA collected and arrange data. All work was performed under supervision of SH. All authors read and approved the final manuscript.

## Authors' information

Bushra Ijaz (M Phil Molecular Biology), Waqar Ahmad (M Phil Chemistry), and Gull S (MSc Biochemistry) are Research Officer at CEMB. Shah Jahan, Saba Khaliq and Samrin A (PhD in Molecular biology), Javed FT is pathologist at Jinnah hospital Lahore. Sarwar MT, Kausar H, Asad S and Shahid I are PhD scholars; while Sajida Hassan (PhD Molecular Biology) is Principal Investigator at CEMB, University of the Punjab, Lahore.

## Pre-publication history

The pre-publication history for this paper can be accessed here:

http://www.biomedcentral.com/1471-230X/11/44/prepub
